# Facial Nerve Palsy in COVID-19-Associated Mucormycosis Patients: A Case Series

**DOI:** 10.7759/cureus.19208

**Published:** 2021-11-02

**Authors:** Rupa Mehta, Nitin M Nagarkar, Krishna Sasanka KSBS, Sree Sudha TY, Ripu Daman Arora, Aakash Aggarwal

**Affiliations:** 1 Otolaryngology, All India Institute of Medical Sciences, Raipur, Raipur, IND; 2 Otolaryngology, All India Institute of Medical Sciences, Deoghar, Deoghar, IND; 3 Pharmacology, Shri Shankaracharya Institute of Medical Sciences, Bhilai, IND

**Keywords:** diabetes, mucormycosis, facial nerve palsy, sinonasal orbito cerebral mucormycosis, covid-19

## Abstract

Sinonasal mucormycosis is a deadly fungal illness that primarily affects diabetics who are uncontrolled. Numerous cranial nerves can be involved; however, facial nerve palsy has only been observed in a few cases. The main objective of this research is to highlight facial nerve involvement as a clinical sign of sinonasal mucormycosis. Nasal stuffiness, headaches, eye pain, orbital edema, ophthalmoplegia, and vision loss are common symptoms in these mucormycosis patients. The study was done in the Department of Otolaryngology & Head and Neck Surgery, All India Institute of Medical Sciences (AIIMS), Raipur, India. Nevertheless, 17 patients with facial nerve palsy (lower motor nerve palsy) and sinonasal mucormycosis arrived at our department. All patients were diabetic, and a majority of patients got Schirmer's test positive with severe stage. In the case of mucormycosis, facial nerve palsy is an unusual but noteworthy symptom. This could be misinterpreted as a cerebrovascular accident (CVA), causing the therapy to be delayed. This is critical as early identification, surgical debridement, and adequate therapy of the underlying metabolic imbalance, as well as amphotericin B, are critical for a successful treatment outcome in mucormycosis.

## Introduction

Sinonasal cerebral mucormycosis (SNCM) is an opportunistic fulminant fungal infection caused by Rhizopus species of the order Mucorales. Baker RD, an American pathologist, created the word "mucormycosis." According to the literature, the annual incidence of mucormycosis is around 1.7 cases per 1,000,000 people [1]. It presents primarily in immunocompromised populations. The most common presentation is SNCM, which accounts for 30%-50% of cases [2]. Within days, nasal mucormycosis aggressively progresses to involve orbits, brain, and skull base [2]. Nasal stuffiness, headache, retro-orbital pain, orbital edema, and slight loss of vision are all common symptoms of SNCM in uncontrolled diabetics or immunocompromised patients. The disease can take six presentations depending on the affected site such as rhinocerebral, orbital, pulmonary, gastrointestinal, cutaneous, and disseminated. Only a few isolated case reports have mentioned facial nerve palsy as a presenting symptom [3,4]. The actual cause of facial nerve palsy is unknown, and no significant facial nerve pathology has been found. However, 17 patients with lower motor neuron facial palsy presented to us. Very few studies are published in this area, so the present study will add valuable information and support further research in this area. This case series intends to raise awareness of this unusual SNCM presenting with facial nerve palsy among ENT surgeons. Additionally, it pretends to explore whether facial nerve palsy is a presenting feature of mucormycosis.

## Materials and methods

Materials and methods

The study was conducted in the All India Institute of Medical Sciences (AIIMS), Raipur, India. All mucormycosis cases admitted between April 2021 and July 2021 were screened for facial nerve palsy. The categorical data has been taken in this study. Patients with facial nerve palsy were recruited and followed by conducting facial topodiagnostic tests. Informed written consents were obtained, and the study was approved by the Institutional Ethics Committee and the letter correspondence number was 1828/IEC-AIIMSRPR/2021.

Case series

A total of 196 mucormycosis patients were presented, in which 25 cases had sino-naso-orbito-cerebral involvement, 46 had sino-naso-palatal, 57 cases had only sino-nasal, and 68 cases had sino-naso-orbital involvement. Out of 196 patients, 17 patients presented with a facial lower motor neuron (LMN) nerve palsy. Age ranges from 30 to 70 years (mean = 48 years), including nine men and eight females, ptosis was observed in five patients, restricted eyeball movements in six patients, vision loss in four patients, and cheek involvement in six patients. All 17 patients were diabetic, of which six were hypertensives. The average time gap between recovery from COVID and onset of mucormycosis symptoms is two to five weeks. Four patients did not have a COVID history. All patients had lower motor nerve palsy in different grades of severity (Table 1). Thirteen out of the 17 patients had right-sided facial palsy, while four had left-sided facial palsy. On Schirmer's test examination, 13/17 patients had severe dry eyes; 12 patients had absent acoustic reflex, and 14 patients had intact taste sensation. All patients were diabetic. Inflammatory markers were raised in almost all patients.

**Table 1:** Severity and grading* of facial nerve palsy in COVID-associated mucormycosis *House-Brackmann grading of facial nerve palsy. DM, Diabetes mellitus; HTN, hypertension; R, right; L, left; LMN, lower motor neuron; Y, Yes; N, No; M, male; F, female.

S. No (Case)	Age	Sex	Comorbidities	Time for the Onset of Mucormycosis Symptoms	Facial Nerve Palsy					
					Side	Type	Grade	Taste	Stape-Dial Reflex	Schirmer’s Test
1	44	F	DM and HTN	2 weeks	L	LMN	5	Yes	N	Severe
2	61	M	DM and HTN	5 days	L	LMN	2	Yes	N	Moderate
3	68	M	DM and HTN	2 weeks	R	LMN	3	Yes	N	Severe
4	42	F	DM	2 weeks	R	LMN	5	Yes	Y	Severe
5	46	F	DM and HTN	6 days	R	LMN	4	Yes	N	Normal
6	45	F	DM	No COVID	L	LMN	4	Yes	N	Severe
7	47	M	DM	1 month	R	LMN	3	Yes	Y	Severe
8	44	M	DM	5 weeks	R	LMN	4	Yes	N	Severe
9	30	M	DM	2 weeks	R	LMN	2	Yes	N	Normal
10	55	F	DM	No COVID	L	LMN	3	Yes	Y	Severe
11	56	M	DM and HTN	2 weeks	L	LMN	2	Yes	N	Severe
12	43	M	DM	No COVID	R	LMN	3	Yes	N	Severe
13	45	F	DM	1 week	R	LMN	5	No	N	Severe
14	52	F	DM	3 weeks	R	LMN	5	Yes	Y	Severe
15	28	M	DM	1 week	R	LMN	2	Yes	Y	Mild
16	64	M	DM	No COVID	R	LMN	4	No	N	Severe
17	46	F	DM and HTN	3 weeks	L	LMN	3	No	N	Severe

Except for three patients, all had intact taste sensation. Schirmer's test score was severe in the majority of the patients. In all the 17 patients, the clinical association between the stapedial reflex (on ipsilateral stimulation) and facial nerve function was studied to see whether the facial nerve was involved in post-COVID with mucor sequel. Thirteen of the 17 subjects had right-sided facial palsy, while four had left-sided facial palsy. Thirteen patients identified severe grades on Schirmer's test. In conclusion, 12 of the 17 subjects had no acoustic reflex. The stapedius (acoustic) reflex (SR) test can help determine where facial nerve damage has occurred. In the therapy of facial nerve palsy, the restoration of the SR (acoustic) to normal after an injury is a good prognostic marker. Inflammatory markers (LDH, S. ferritin, D-dimer, and CRP) were raised in most of the patients.

Radiologically, pterygopalatine and infratemporal fossa involvement was seen in 10 cases, while premaxillary space involvement was seen in four cases. Fifteen patients had a combined approach of surgical debridement (maxillectomy, ethmoidectomy, sphenoidotomy, and frontal sinusotomy), three patients had a craniotomy, temporal lobe abscess clearance by a neurosurgeon, and the remaining three had orbital exenteration. Intraoperatively premaxillary space involvement was seen in seven patients, and pterygopalatine and infratemporal fossa involvement was seen in 11 patients. All the patients were treated with liposomal amphotericin B with a total cumulative dose of around 2-2.5 g depending upon the severity of the infection.

## Results

**Figure 1:**
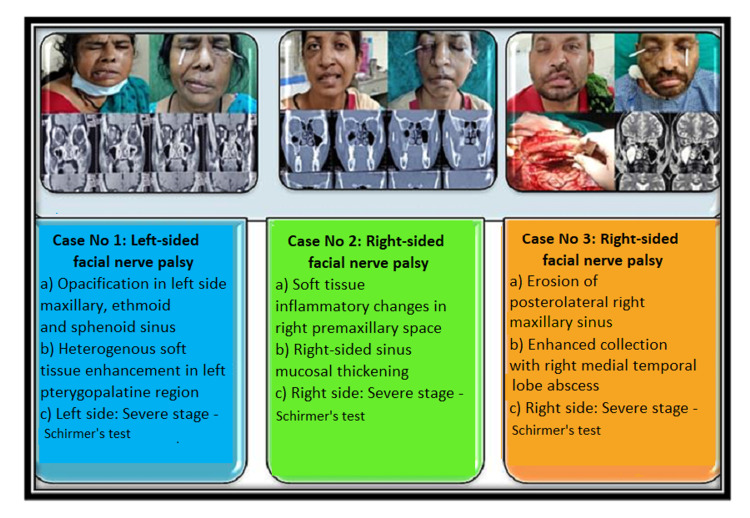
Presentations of facial nerve palsy in mucormycosis patients (Cases 1-3)

**Figure 2:**
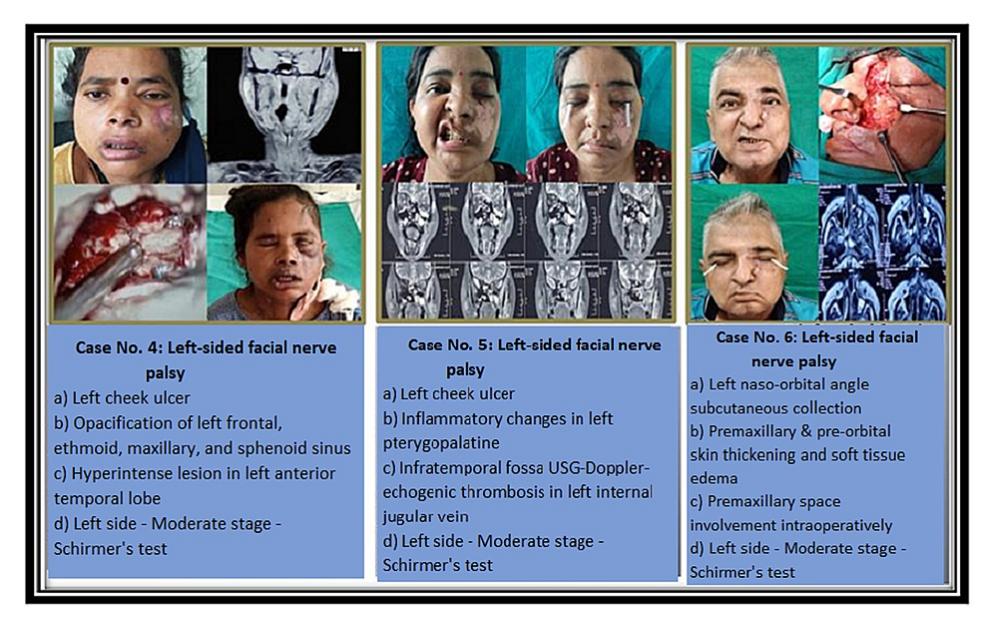
Presentations of facial nerve palsy in mucormycosis patients (Cases 4-6)

Presentations of facial nerve palsy in mucormycosis patients (Cases 1-6) are shown in Figures 1, 2.

**Figure 3:**
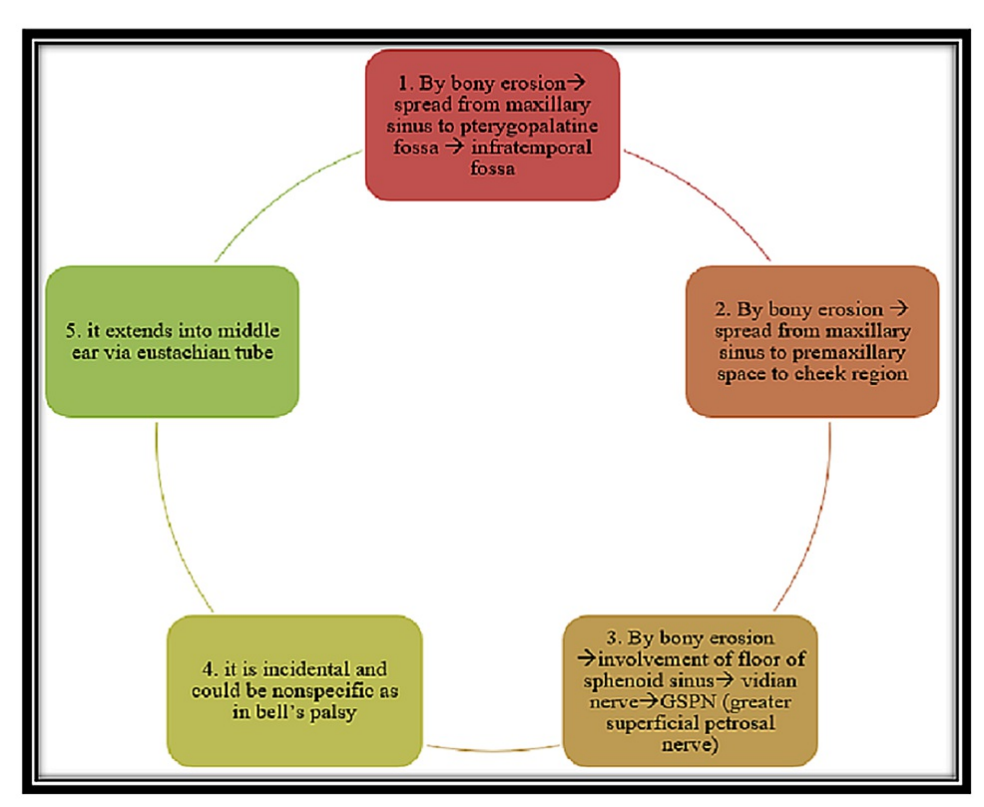
Hypothesis of facial nerve palsy in COVID-associated mucormycosis GSPN, Greater superficial petrosal nerve.

 The hypothesis of facial nerve palsy in COVID-associated mucormycosis is shown in Figure 3.

## Discussion

Mucormycosis is an opportunistic infection that can be fatal in patients with metabolic acidosis, iron overload, immune deficiency syndrome, organ transplant, fasting, burns, and those on steroids or deferoxamine [5,6]. All of these factors influence the development of hyphae and the germination of spores [7]. COVID-19 is a major trigger that raises the risk of mucormycosis. Mucormycosis became an epidemic in the pandemic of COVID-19. In India, places like Maharashtra, Gujarat, Madhya Pradesh, Haryana, Delhi, Uttar Pradesh, Bihar, Chhattisgarh, Karnataka, and Telangana were all affected with mucormycosis. Although facial nerve paralysis was not one of the commonest symptoms, it was occasionally seen (Figures 1, 2).

A total of 60% of all cases and 90% of rhinocerebral cases are caused by *Rhizopus oryzae*. The involvement of many tissues in the rhinocerebral area might cause clinical symptoms to reflect as cerebrovascular accidents (CVA) [6]. The pathophysiology of mucormycosis is caused by fungal spores that enter the host through inhalation. Germination of these spores produces hyphae that invade the blood vessels, causing thrombosis, which finally obstructs the veins and causes ischemia. This results in the black necrotic eschar on which the fungus feeds, causing infection to spread quickly across the surrounding area [8]. The fungus reaches the nasal cavity and spreads to the sinus cavities through the ethmoidal, angular, and lacrimal arteries to invade the palate, orbit, and intracranially [9]. Meningitis, cavernous sinus thrombosis, frontal and medial temporal abscess, and facial nerve paralysis are all presenting features of sino-naso-orbito cerebral mucormycosis. The actual cause of facial nerve palsy and its pathology is unknown. A few authors reported (Figure 3) that the nerve exiting the stylomastoid foramen may be affected by bony erosion to the infratemporal fossa via the maxillary sinus to the pterygopalatine fossa. The pterygopalatine fossa is also thought to be a mucor reservoir from which mucor extends to the orbit's retroglobal and infratemporal spaces [9]. As a result, a pterygopalatine fossa infection can extend to the orbital apex, inferior orbital fissure, and infratemporal fossa. Mucorales species have been found to migrate along peripheral nerves in some research studies [6]. The proximity of the pterygopalatine fossa to the cranial tissues as well as the presence of numerous lineages of vascular and neural tissue makes it a plausible route of perineural invasion [6]. Another hypothesis is that the facial nerve palsy is incidental and could be nonspecific as in Bell’s palsy. There were case reports of mucormycosis extending to the middle ear via the Eustachian tube and the facial nerve [5]. However, this was not observed in our case series as only two cases had ear (opposite side of facial palsy) discharge (mucosal COM) for so many years. Involvement of the floor of sphenoid sinus via the Vidian nerve, which is a continuation of the greater superficial petrosal nerve (GSPN) is also a viable alternative [10].

Along the nerve sheath of the intracanalicular facial nerve within the temporal bone, a solitary involvement can occur [11]. As demonstrated in the current study of patients, this explains facial nerve affection without the involvement of any other cranial nerves. An additional cause of facial nerve palsy in people with diabetes is the disease of the resistance arteries, which can produce edema and localize facial nerve ischemia. This would impair the nerve's blood supply, resulting in palsy [12]. Garcin syndrome is defined as the involvement of more than seven cranial nerves [13] on the same side without elevated intracranial tension or sensorimotor symptoms. One study described a case of Garcin syndrome in mucormycosis, in which the patient had long tract symptoms as a result of a blockage of the internal carotid artery. This illness is extremely uncommon and is caused by malignancies of the skull base as well as primary nasopharyngeal cancer. Tuberculous meningitis resembles rhinocerebral mucormycosis in that the former frequently manifests as numerous cranial palsies [14]. Only four patients out of 17 showed six cranial nerve palsies (second, third, fourth, fifth, sixth, and seventh CN) indicating that Garcin syndrome was not evident in our patients. Because of the inadequate drug delivery to the lesion due to significant arterial thrombosis, medical therapy alone is ineffective [15]. If the patient responds to amphotericin, oral posaconazole can be administered instead, although it is not indicated as a first-line treatment [16]. The consequence of facial nerve palsy and the prognosis of patients is yet to be noticed and cannot be confirmed from our case series. Schirmer's test was ideal for determining whether the facial nerve was involved. In our study, the majority of the patients with facial palsy have been affected by GSPN involvement. This study is an observational case series of patients with mucormycosis and facial palsy as the initial presentation. It is interesting because it is a rare disease, and facial palsy is indeed an unusual presentation. Our concern is there are very few studies published about this topic. Moreover, the incidence of mucormycosis seems to be rising, particularly in India.

## Conclusions

Mucormycosis is a fungal infection that spreads quickly and is lethal. The cornerstone to a patient's survival is timely diagnosis and treatment. Mucormycosis can manifest with facial nerve palsy; in such cases, it might be mistaken as CVA and enabling therapy might be delayed. The authors' intent in this study is to stress facial nerve paresis as a presenting symptom of rhinocerebral mucormycosis. Antifungal medicines, reversing of exacerbating potential risks, and prompt surgical debridement are all part of the treatment. In our series, the prognosis for the restoration of facial nerve function is still not excellent, and more time is needed for follow-up. The influence of facial nerve palsy on the outcomes of mucormycosis patients is yet to be established and cannot be inferred from a short case series. For this, we recommend conducting a comprehensive study involving larger case series of mucormycosis.
